# Do Nectar Guides in Flowers of Two *Myosotis* L. Species (Boraginaceae) Serve as Osmophores?

**DOI:** 10.3390/plants15172658

**Published:** 2026-08-30

**Authors:** Elżbieta Weryszko-Chmielewska, Aneta Sulborska-Różycka, Marta Dmitruk

**Affiliations:** Department of Botany and Plant Physiology, University of Life Sciences in Lublin, Akademicka 15, 20-950 Lublin, Poland

**Keywords:** borage, forget-me-not, visual attractants, petal structure, scent glands, faucal

## Abstract

Osmophores, which constitute scent-releasing glands in flowers, have so far been insufficiently characterised. This study is the first to present the structure of scent glands exhibiting features consistent with those of osmophores in two *Myosotis* species (Boraginaceae). Petal tissues of *Myosotis scorpioides* and *M. sylvatica* were analysed using light microscopy, scanning electron microscopy, and transmission electron microscopy. Histochemical tests were performed to detect fragrance-producing tissues (neutral red), phenols (Toluidine blue O), lipids (Sudan III, Sudan Red B), and starch (Lugol solution). The study has shown that the epidermal cells within the yellow nectar guides (faucal scales) were differentiated, as they comprised palisade-like cells, papillae, and unicellular trichomes. The petal appendages forming white ridges on the petals were covered with papillae. Histochemical assays revealed the presence of scent-producing epidermal cells (palisade-like, papillae, unicellular trichomes), lipids (which is typical of scent glands) and starch during the pre-secretory phase. The ultrastructure of the examined cells exhibited dense cytoplasm, large nuclei, and numerous mitochondria and plastids. Such organelle composition is associated with secretory activity, which is further supported by the presence of numerous secretory vesicles located in the vicinity of the plasmalemma, releasing their contents into the periplasmic space. The histochemical analyses and the observations of the anatomical structure and ultrastructure of the cells indicated that both the faucal scales and the petal appendages exhibit features of osmophores. These findings contribute to expanding the extant knowledge on the structure of scent glands with traits typical of osmophores and the characteristics of the Boraginaceae family.

## 1. Introduction

### 1.1. Multimodal Floral Cues

In flowers of animal-pollinated plants, multiple cues are involved in attracting floral visitors. These include the colour, scent, flower size, shape, symmetry, orientation, nectar guides, and texture [1,2,3,4,5], as well as the humidity of petals, temperature, and electrical field [6]. Multimodal signals are a combination of different sensory cues that help pollinators to find rewards, mainly nectar and pollen, as well as resin and oil [7]. The cues essential for insects and other pollinators include colour and scent. These traits enable insects to find flowers even from considerable distances and are, therefore, also referred to as signals [1]. Floral volatiles have been demonstrated to be particularly effective in helping pollinators find flowers over long distances [8]. Plants’ volatile organic compounds can also play other roles, such as in defence against herbivores and predators, plant-to-plant communication, thermo-tolerance, and adaptation to environmental stress [9].

The volatile compounds contained in flower essential oil that are highly effective at attracting pollinators are represented by terpenes and terpenoids, including linalool, limonene, myrcene, geraniol, cineole, and various pinene compounds [10]. These metabolites are most often characterised by sweet floral scents, e.g., geraniol has a rose-like scent, limonene exhibits a citrus scent, and linalool has a lavender scent. Linalool is present in nearly all floral volatiles, and geraniol is a powerful attractant for bees, enabling these insects to navigate bee pastures. Interestingly, different odours of volatiles originating from different flowers attract the same types of pollinators [9]. Visual cues, including petal colour, ultraviolet patterns, and nectar guides, provide insects with information about landing sites and food location [2,11]. Petal warmth and texture facilitate the identification of flowers and the assessment of reward levels [1,6].

### 1.2. Nectar Guides

Nectar guides are special colour marks within the corolla that are associated with the location of food in flowers. They create a high chromatic contrast against the rest of petals and, hence, become attractive to different insect pollinators. Nectar guides enable pollinators to find food rewards in flowers and act as short-distance cues [2].

In zygomorphic flowers, nectar guides are situated on different petals. They constitute one system consisting of radiating lines, spots, stripes, dashes, or irregular patterns pointing toward the centre of the flower. They guide the last steps of the landing for pollinators, e.g., in the flowers of Lamiaceae [2].

In radially symmetrical flowers, the nectar guides are concentric and circular, such as in the yellow or white throat, contrasting with the blue petals of *Myosotis* flowers [2,12]. Nectar guides have been shown to emit scents of varied quality with respect to other petals [13].

Floral volatiles are usually a result of multiple scents developed in the perianth, stamens, pollen, pistils, and nectaries. However, the petals are the main source of volatiles [4,14,15,16,17].

In the flower scent mixture, certain volatiles are attractive to pollinators, and some of them protect the flowers from destructive animals, pathogens, herbivores, and other enemies [7].

### 1.3. Osmophores

In certain plants, volatile organic compounds are produced in only specified sites in flowers and inflorescences, containing specialised glands, i.e., osmophores [18,19]. They may constitute part of the perianth, bracts, appendages of peduncles or anthers [18,19,20]. The surfaces of osmophores are smooth, covered with papillae or different types of trichomes [18,19,21], and often wrinkled or rugose, which facilitates scent emission [22]. Osmophores can be formed from a single epidermis layer, or from the epidermis and a few layers of subepidermal parenchyma [18,19].

So far, osmophores have been identified in many species of Orchidaceae [22,23,24], Araceae [18,25], Gesneriaceae, Solanaceae, Euphorbiaceae [26], Anacardiaceae [27], Podostemaceae [28], and Malpighiaceae [29], as well as in plants from other families. Recent publications (2023–2026) on osmophores address their location, structure, interaction with specific pollinator species, and their evolutionary roles in pollination. In their study on *Ophrys speculum* (Orchidaceae), Francisco and Ascensão [30] presented new evidence of mimicry in this species. They determined the location of osmophores in the apical marginal zone of the labellum. The scent emitted by osmophore tissues and the visual and micromorphological features were shown to be involved in specific sexual interactions with scoliid wasps. Paiva et al. [25] investigated osmophores on *Calladium bicolor* (Araceae), and identified the distal zone of the staminate part of the spadix as a functional osmophore responsible for thermogenesis and the emission of a scent that attracts beetles. The tissues forming the osmophore consisted of a papillate epidermis with a stomata and an underlying secretory parenchyma containing starch grains in its cells. The release of heat accompanying scent emission facilitates scent dispersion. Macedo et al. [31] conducted extensive studies on the location, anatomy, and histochemistry of 18 species of the Bignonieae tribe (Bignoniaceae). The researchers identified six different patterns of osmophore distribution in the corolla tubes and lobes. They also found that 94% of bee-pollinated species exhibited a papillose epidermis, while the bird-pollinated species had a flat epidermis. As reported by Possobom and Machado [29], the osmophores in three Neotropical Malpigiaceae species exhibited structural and functional differences. A scent-emitting zone located at the base of the petals was observed in *Byrsonima coccolobifolia* flowers, while osmophores in the other two species (*Banisteriopsis variabilis* and *Peixotoa reticulata*) were located along fimbriated petal margins. The shapes of cells in the osmophore epidermis varied, ranging from cone-shaped to rounded.

### 1.4. Traits of Boraginaceae Flowers

The Boraginaceae comprises 154 accepted genera, whereas 156 species are reported within the genus *Myosotis* L. [32,33,34,35].

The inflorescence in Boraginaceae is usually a scorpioid cyme. In *Myosotis*, the inflorescence is a true scorpioid cyme (cincinnus), in which lateral branches arise on one side of the main shoot [36]. Boraginaceae flowers are bisexual, pentamerous, and hypogynous. The biseriate perianth is mostly radial. A typical trait is the great diversity in the corolla form, colour, and size. The corolla, composed of fused petals, is often tubular or campanulate and of various lengths [37]. A very short tube with broad flat lobes is observed in *Myosotis* spp., whereas a much longer tube is exhibited by *Symphytum officinale* [38]. This family is characterised by considerable variation in flower size. Very small flowers, with corollas measuring only 1 mm in length, are produced by *Cryptantha* and *Microcaryum*, whereas the corolla in *Lithospermum* can reach >90 mm in length. The corolla in Boraginaceae is commonly blue, purple, pink, or white. Lithospermeae plants produce orange and yellow corollas, while the corollas in Boragineae may also be yellow. In many species in this family (e.g., *Symphytum*, *Pulmonaria*), the corolla changes colour from pink to blue and dark purple [37].

The peculiar features observed in the architecture of the Boraginaceae corolla include basal and faucal scales. Basal scales arise internally in the lower part of the corolla tube, whereas faucal scales (fornices) occur around the throat of the tube. These usually contrastingly coloured structures, compared to the corolla lobes, restrict access to the corolla tube. Faucal scales occur in the majority of Boraginaceae taxa, but they are most often absent in genera producing tubular flowers. Many species in this family also have basal scales, which are sometimes reduced to small folds [37].

Among the 28 species of the family Boraginaceae reported by Jeiter et al. [39], 16 species (e.g., in the genera *Anchusa*, *Symphytum*, *Borago*, *Alcanna*, *Lithospermum*, and *Cynoglossum*) were found to possess both basal and faucal scales, 10 species (e.g., in the genera *Cerinthe*, *Onosma*, *Echium*, and *Glandora*) had only basal scales, only one species (*Amsinckia*) had exclusively faucal scales, and one species (*Moltkia*) of this family had no scales, which seems to suggest that the absence of fornices in the corolla is rare in the Boraginaceae family. Other studies have indicated that both basal and faucal scales occur within the corolla tube of *Myosotis* spp. flowers [38,40]. The campanulate corolla in *Symphytum* has five large, triangular faucal scales. However, some of the smallest faucal scales are observed in the flowers of *Borago pygmaea* [39]. These authors reported the presence of bilobate faucal scales, similar in shape to those found in *Myosotis*, *Nonnea*, *Lithospermum*, and *Cynoglossum*.

Faucal and basal scales differ in shape and size among species within the family [39]. Basal scales may form a ring-shaped intrusion or occur as free scales. In turn, faucal scales form a ring around the opening of the tube, and may be papillose or pubescent [37]. Basal scales sometimes cover the nectary and ovary, as observed in the flowers of *Myosotis sylvatica* [40].

A characteristic feature of the floral structure in Boraginaceae is the position of stamens, which adhere to the corolla, forming a stamen–corolla tube [39], and are fused with the tube to a different extent. In representatives of the genus *Myosotis*, connective extensions in the stamens form fleshy appendages [41]. The pistil is composed of two carpels. The superior ovary is four-lobed due to the presence of two false septa. The style is single, with a capitate to oblong stigma [37]. The nectary, in the form of a disc, is located at the base of the ovary [42,43].

The present study is a continuation of our previous research on the floral structures of *Myosotis scorpioides* and *M. sylvatica*. So far, we have focused on the ultrastructure of pollen grains and the characteristics of floral nectaries analysed at several levels of organisation [40,41,43]. *Myosotis scorpioides* has exhibited morphological variability and certain similarities to *M. sylvatica*, with which it forms hybrids. The main distinguishing features between these two species include the position and form of non-glandular trichomes on the calyx. In *M. sylvatica,* they are protruding and hooked, whereas in *M. scorpioides*, they are straight and adhering [44].

In view of the above findings, this study aimed to test the hypothesis that faucal scales and petal appendages in *Myosotis scorpioides* and *M. sylvatica* flowers not only function as nectar guides, but may also serve as osmophores. Its specific objective was to characterise the detailed structure of different parts of petals in the flowers of the examined plants. These analyses focused on the micromorphology of the petal surface, petal anatomy, and the ultrastructure of faucal scales located at the entrance to the corolla tube. The study also aimed to address the following questions:

(i) How are the petals structured in the region where faucal scales are found?

(ii) What is the surface structure of the faucal scales, petal appendages, and the remaining parts of the petals?

(iii) Do the histochemical features of the examined epidermal cells correspond to the properties of secretory tissues?

(iv) Do the anatomy of the faucal scales and petal appendages and the ultrastructural traits of the epidermal cells of the faucal scales indicate secretory properties typical of osmophores?

## 2. Results

### 2.1. Visual Attractants of Flowers

**Figure 1:**
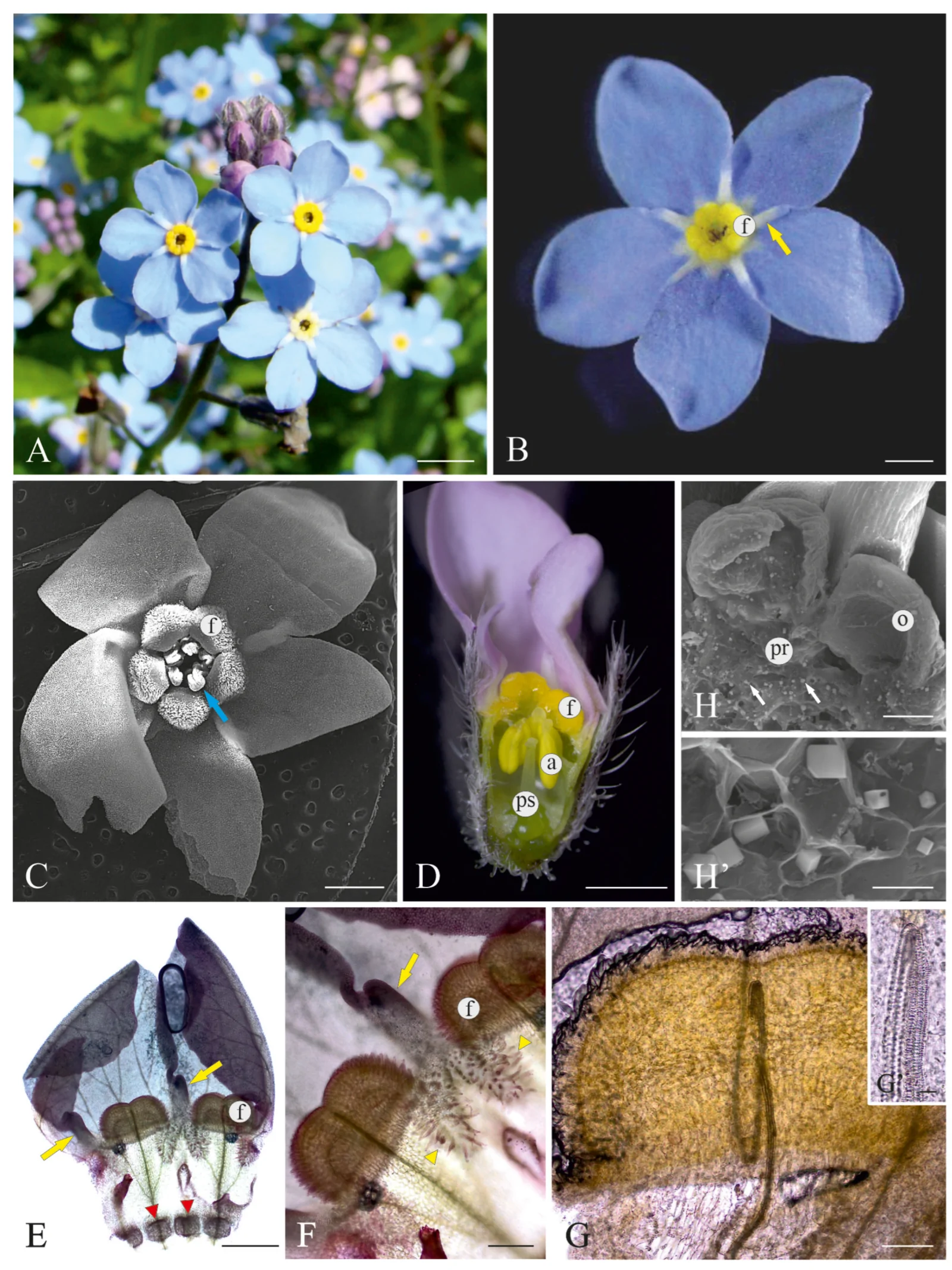
**Inflorescences and flowers of *M. scorpioides* (C,H,H’) and *M. sylvatica* (A,B,D,E–G’); stereoscopic microscopy (SM)—(B,D), scanning electron microscopy (SEM)—(C,H,H’), light microscopy (LM)—(E–G’).** (**A**) Flowers borne on scorpioid cymes. (**B**) Magnification of a flower with visible faucal scales and white appendages on petals (yellow arrow). (**C**) Flower with velvety texture of petals; blue arrow shows an appendage of stamen connectives. (**D**) Faucal scales and anthers on the longitudinal section of a flower. (**E**) Inner part of the flower with faucal scales, appendages on petals (yellow arrows), and basal scales (red arrowheads). (**F**) Parts of petals with faucal scales, appendages on petals (yellow arrows), and trichomes beneath them (yellow arrowheads). (**G**) Faucal scale with a vascular bundle running through its centre. (**G’**) Magnification of a vascular bundle of the faucal scale with helical vessels. (**H**) Part of the longitudinal section of a flower with the ovary and receptacle parenchyma with visible calcium oxalate crystals (white arrows). (**H’**) Magnification of receptacle parenchyma cells with prismatic calcium oxalate crystals. (**F**,**G**) Staining with Sudan Red B; a—anther, f—faucal scale, o—ovary, ps—pistil. Scale bars = 3 mm (**A**); 1 mm (**B**,**D**); 0.7 mm (**C**); 0.5 mm (**F**); 200 µm (**G**); 100 µm (**E**,**H**), 50 µm (**H’**), 5 µm (**G’**).

The flowers of *Myosotis scorpioides* and *M. sylvatica* exhibited age-dependent colouration. In both species, flowers at the bud stage were pink; during the first days of anthesis, their petals were intensely blue (Figure 1A,B), and at the end of flowering (days 3–4), they turned light blue (Figure 1A).

Colour changes occurring during anthesis also affected the yellow ring of faucal scales located in the central part of the flower, which functioned as a nectar guide. In newly opened flowers, the faucal scales were intensely yellow. The yellow colour became paler on subsequent days and, at the end of flowering, these structures turned whitish (Figure 1A). Moreover, on the last day of anthesis, brown necrotic spots emerged within the faucal scales.

Regardless of flower age, white appendages in the form of short ridges were visible at the petal margins near the corolla tube (Figure 1A,B).

The faucal scales constricted the throat of the corolla tube and covered the stamens located beneath them. Only the appendages of stamen connectives were visible from above through the formed narrow opening (Figure 1C). Longitudinal sections of the flower also revealed the position of anthers within the corolla tube (Figure 1D). The positions of faucal scales, basal scales, and petal appendages were visible after cutting and spreading a fragment of the corolla (Figure 1E). The extension from the appendages toward the corolla tube revealed two clusters of long trichomes (Figure 1F). Each faucal scale was supplied by a single vascular bundle running centrally. The bundle had a well-developed xylem revealing four strands of helical vessels (Figure 1G,G’). Furthermore, prismatic calcium oxalate crystals were observed in the parenchyma cells of the floral receptacle region (Figure 1H,H’).

### 2.2. Micromorphology of Petals

**Figure 2:**
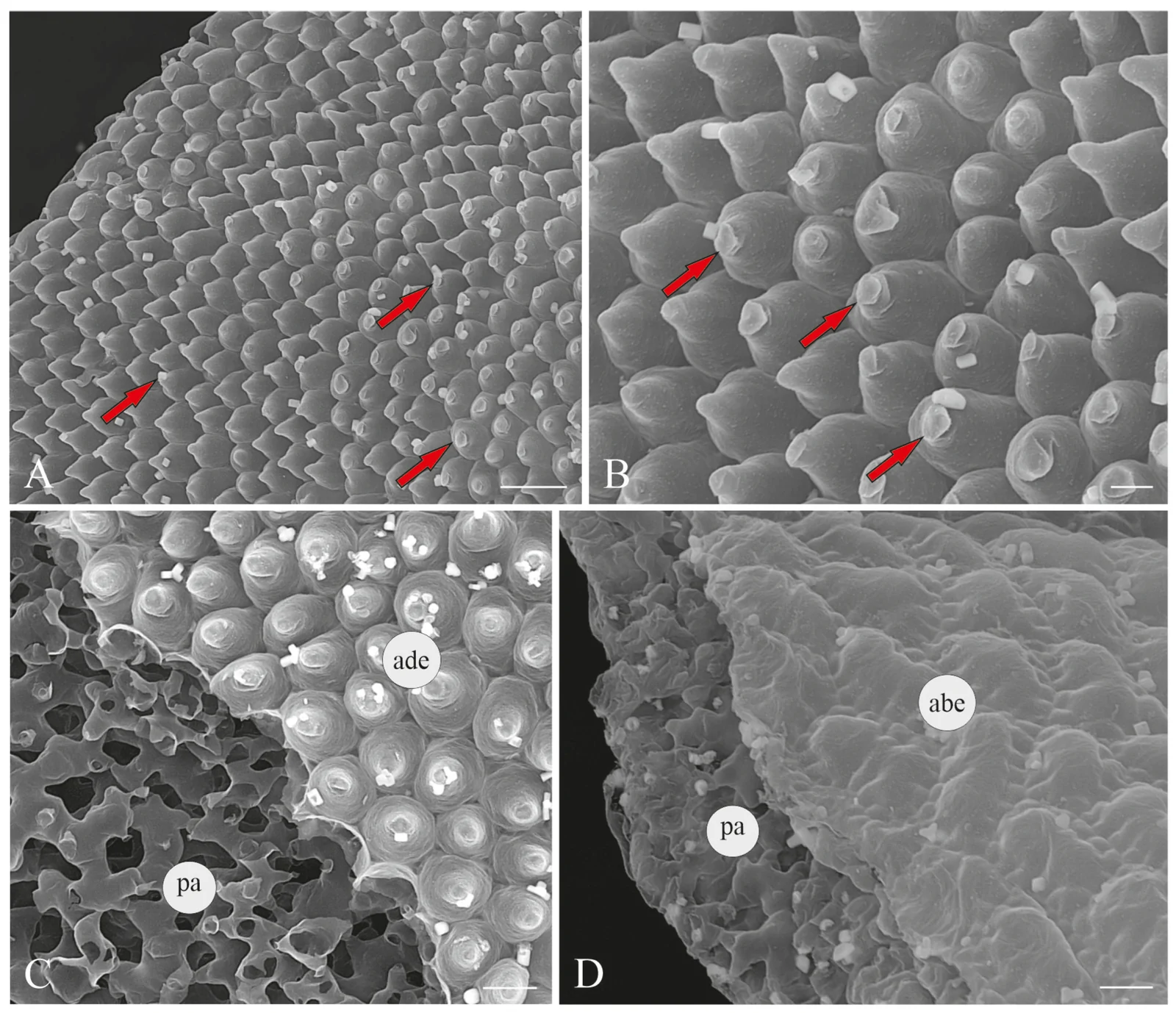
**Micromorphology of petals of *M. scorpioides* visible in SEM.** (**A**,**B**) Adaxial surface of the petal with conical papillae, in the top part of which there are visible protrusions with secretion (red arrows). (**C**) Conical-papillate epidermal cells on the adaxial surface and parenchyma cells. (**D**) Abaxial surface of the petal and parenchyma; abe—abaxial epidermis, ade—adaxial epidermis, pa—parenchyma. Scale bars = 40 µm (**A**), 20 µm (**C**,**D**), 10 µm (**B**).

SEM analyses of the adaxial petal surface demonstrated the presence of conical papillae (Figure 2A,B). They exhibit a smooth cuticle forming apical protrusions, which are probably filled with secretion. Loosely arranged mesophyll parenchyma cells (Figure 2C) form two to three layers in the petals. The epidermis of the abaxial surface of petals consists of cells with convex external walls and characteristic radial walls showing numerous undulations (Figure 2D).

### 2.3. Micromorphology of Faucal Scales

**Figure 3:**
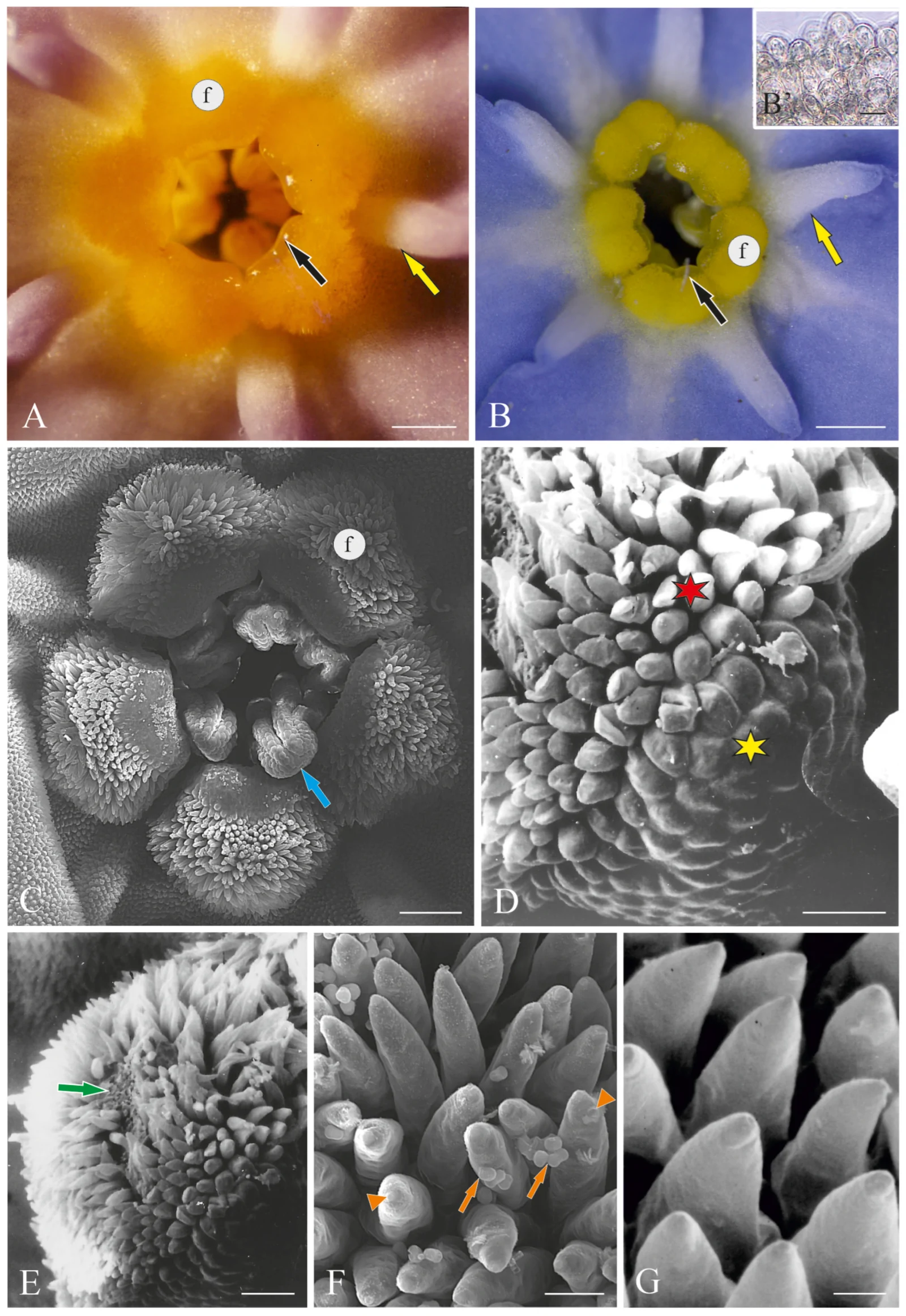
**Morphology of faucal scales and appendages on petals of *M. scorpioides* (A,C–G) and *M. sylvatica* (B); (A,B)—stereoscopic microscopy (SM), (C–G)—scanning electron microscopy (SEM).** (**A**,**B**) Yellow faucal scales in flowers in the top view with a visible entrance to the flower throat and appendages on the petals (yellow arrows); black arrows show the smooth part of putative osmophore. (**B’**) Magnification of a white appendage on the petals with visible papillae. (**C**) Entry to the flower throat with visible faucal scales and appendages of anther connectives (blue arrow). (**D**) Part of the faucal scale with smooth epidermal cells (yellow star) and surface covered with papillae and trichomes (red star). (**E**) Pubescent part of the faucal scale with visible loss of trichomes (green arrow). (**F**) Trichomes on the surface of faucal scale with traces of secretion (orange arrowheads) and pollen grains (orange arrows) on the top. (**G**) Apical parts of trichomes present on the surface of the faucal scale; f—faucal scale. Scale bars = 500 µm (**B**), 300 µm (**A**), 200 µm (**C**), 100 µm (**E**), 50 µm (**D**), 20 µm (**F**), 10 µm (**B’**,**G**).

The position of faucal scales within the flower and their yellow colour contrasting with the blue petals (Figure 3A,B) allow a conclusion that they function as nectar guides in both *Myosotis* species. Each ring of faucal scales consists of five elements touching each other and positioned above the petals. However, the studied species differ in the shape and size of the aforementioned ring elements. In *M. sylvatica*, the ring is composed of five components, each divided into two rounded parts (bilobate) (Figure 3B,C), whereas the components in *M. scorpioides* are slightly wider in the radial direction and lack a noticeable division (undivided) (Figure 3A). The surface of individual ring components is heterogeneous. On the throat-facing side, the surface of the epidermal cells is smooth and slightly glossy (Figure 3A,B), whereas the more external zone is covered with trichomes of various lengths (Figure 3C,D). In some areas, a loss of trichomes can be observed, probably caused by insect feeding (Figure 3E). The surface of the trichomes is smooth (Figure 3F,G), and the SEM observations revealed traces of secretion and pollen grains adhering to the trichomes (Figure 3F).

At a greater magnification of the central flower region, the white appendages in the form of ridges appear as thickenings along the petal margins near the flower centre, between the faucal scales (Figure 1B and Figure 3A,B). They are constituted by parenchyma cells and are covered with epidermal cells forming papillae (Figure 2B).

### 2.4. Anatomy of Faucal Scales

**Figure 4:**
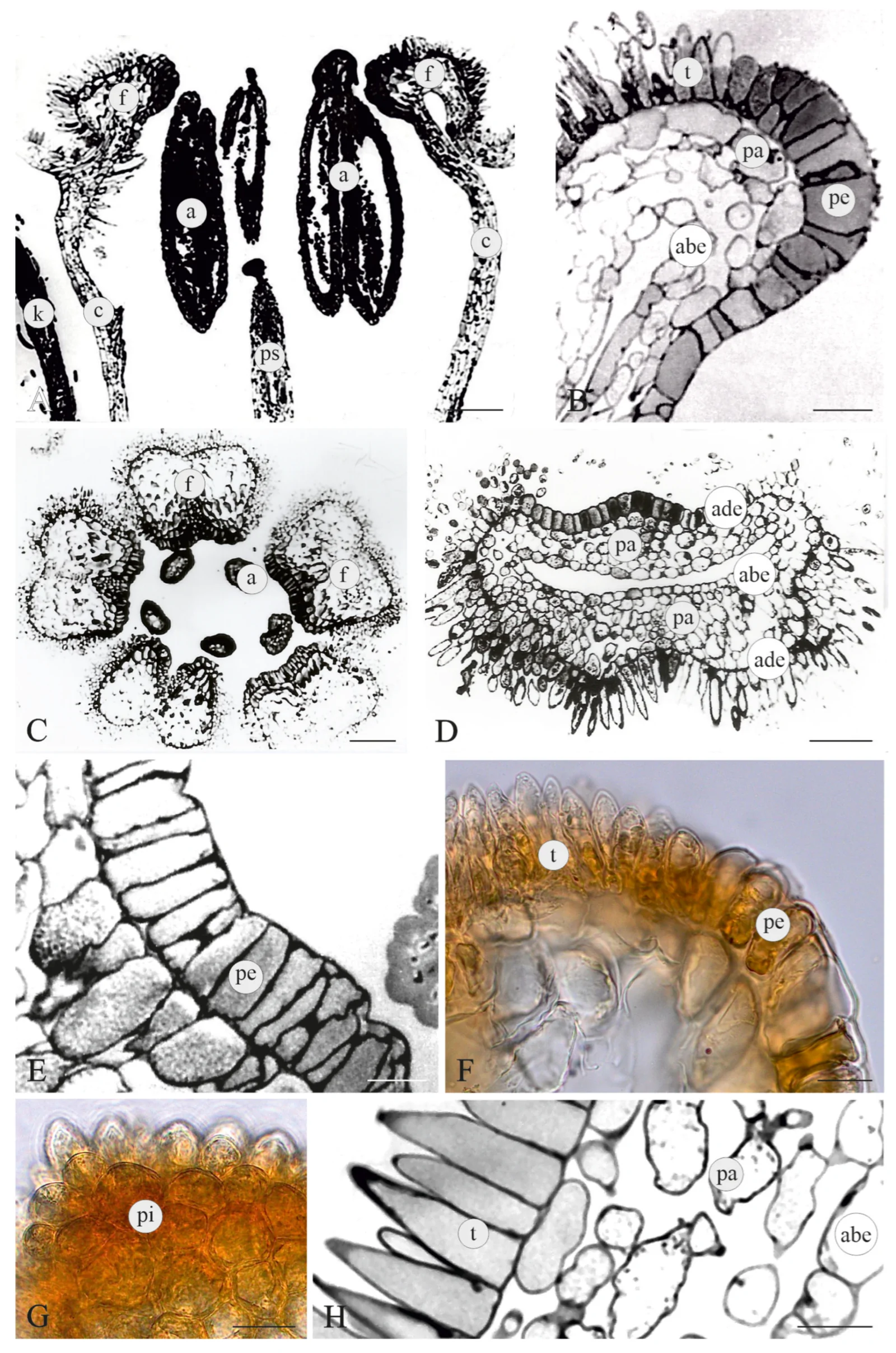
**Anatomy of *M. sylvatica* faucal scales.** (**A**) Part of the longitudinal sections of a flower with visible faucal scales, anthers, and the apical part of pistil. (**B**) Part of the longitudinal section of faucal scale with palisade-like epidermal cells and trichomes. (**C**) Part of the cross-section of a flower with visible faucal scales and anthers. (**D**) Cross-section of the faucal scale. (**E**,**F**) Parts of cross-sections of the faucal scale with palisade-like epidermal cells, papillae (**G**), and trichomes (**H**); a—anther, abe—abaxial epidermis, ade—adaxial epidermis, c—corolla, f—faucal scale, k—calyx, pa—parenchyma, pe—palisade-like epidermal cell, pi—papillae, ps—pistil, t—trichome. Scale bars = 200 µm (**A**,**C**), 100 µm (**D**), 30 µm (**B**), 20 µm (**E**–**H**).

Longitudinal sections clearly showed that the faucal scales significantly constricted the throat of the corolla tube, thereby restricting insect access to the interior of the flower (Figure 4A). The observed scales were formed by petal folding and thickening in the upper part of the corolla tube (Figure 4A,B). Both longitudinal and transverse sections revealed that the epidermal cells oriented inward within the faucal scales are palisade-like (Figure 4B–F), those oriented upward form papillae (Figure 4G), and those adjacent to them are elongated, conical, unicellular trichomes of various length (Figure 4B,D,F,H). The thickness of the petal in the scale region is formed by four to five layers of parenchyma (Figure 4B,D,H), whereas only two to three layers are present in the other petal regions.

### 2.5. Histochemical Tests

**Figure 5:**
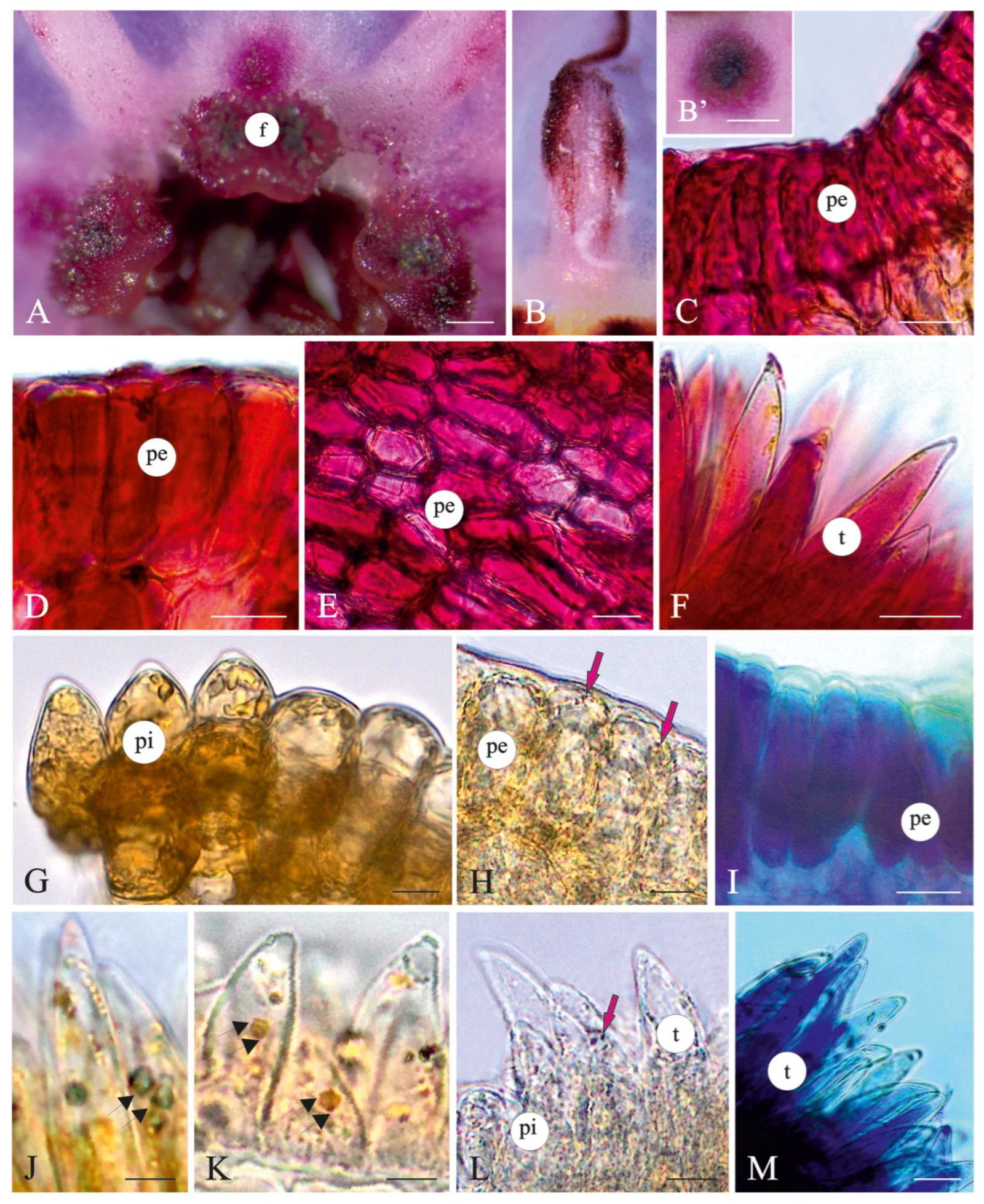
**Histochemical tests.** (**A**–**F**) Reaction with neutral red indicating active scent-producing zones. Intense pink-red staining of faucal scales (**A**), appendages (**B**), circular surfaces on petals (**A**,**B’**), palisade-like epidermal cells in the cross-section (**C**,**D**) and in the top view (**E**), and non-glandular trichomes (**F**). Parts of cross-sections of the faucal scale with palisade-like epidermal cells, papillae (**G**,**H**), and trichomes (**H**–**M**); droplets of secretion are visible in some trichomes (double black arrows); (**J**) control, (**K**) lipid droplets after staining with Sudan III. Starch grains (pink arrows) present in palisade-like epidermal cells (**H**) and trichomes. (**L**) Staining with Lugol solution. Phenolic compounds present in palisade-like epidermal cells (**I**) and trichomes. (**M**) Staining with Toluidine blue O; f—faucal scale, pe—palisade-like epidermal cell, pi—papillae, t—trichome. Scale bars = 300 µm (**B’**), 200 µm (**A**,**B**), 20 µm (**C**–**F**,**I**,**M**), 10 µm (**G**,**H**,**J**–**L**).

After separating the corolla from the rest of the *M. sylvatica* flower and treatment with neutral red, we found that some of its parts were stained with varying intensities of pink and red shades. The most intensely stained structures were the nectar guides (Figure 5A) and parts of the white stripes on the radial edges of the petals (Figure 5B), as well as their fragments located next to the nectar guides, which were circular in shape (Figure 5A,B’). In many flowers, we observed only local pink colouration in different parts of the petals. In the cross-sections of the nectar guides, intense red colouration appeared in the palisade-like epidermis cells (Figure 5C,D), which was also visible in the tangential sections of these cells (Figure 5E). In turn, the vacuoles contained in the trichomes exhibited varying degrees of staining (Figure 5F). The red and pink colouration indicates the presence of vacuoles in fragrance-producing cells.

In *M. sylvatica* fresh flowers, greenish droplets of secretion were observed in palisade-like cells, papillae, and trichomes (Figure 5G,J). Their colour turned orange upon staining with Sudan III, indicating the presence of lipid compounds (Figure 5). The histochemical test for starch detection (Lugol solution) performed on *Myosotis* bud tissues yielded positive results for the palisade-like cells, papillae, and trichomes of faucal scales (Figure 5H,L), as well as for the papillae of appendages. The presence of starch in the pre-secretory stage is typical for secretory cells. The treatment of fresh flower sections with Toluidine blue O revealed the presence of phenolic compounds in all epidermal cells of the putative osmophores (palisade-like epidermis, papillae, trichomes) (Figure 5I,M).

The sensory test performed 30 min after placing flower fragments in plastic containers revealed a nearly imperceptible odour, likewise in the test conducted after 12 h. The results of the sensory test performed after 24 h indicate that the nectar guide zone in *M. sylvatica* flowers emits a substantially stronger odour than the other parts of the petals. On a scale of 1–5, the intensity of the scent from the nectar guides was rated on average at 3, while the blue flower fragments received an average rating of 1. The scent from the nectar guides was described as citrus-floral, while the weak scent from the other parts of the petals could not be determined.

### 2.6. Ultrastructure of Cells of Faucal Scales

**Figure 6:**
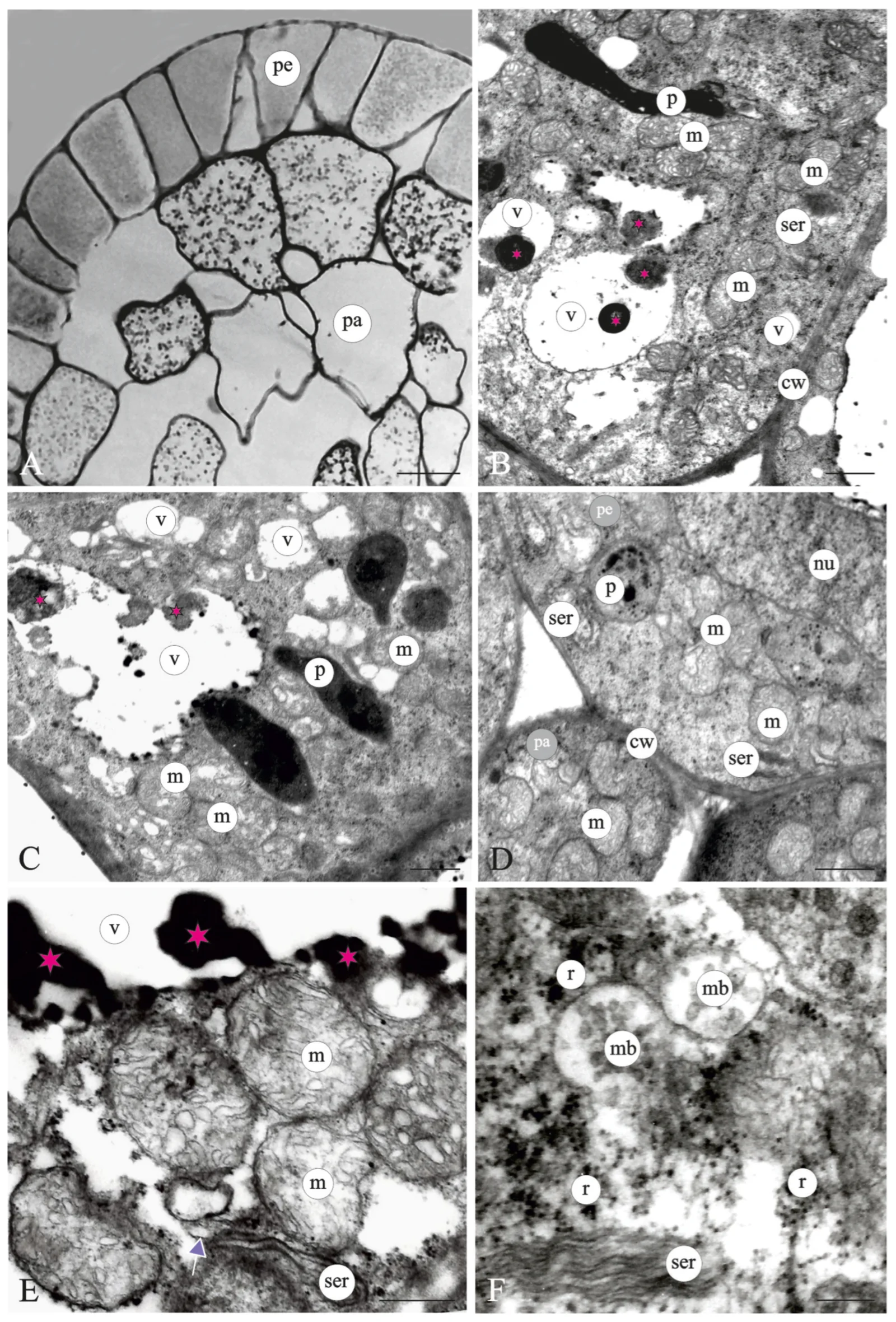
**Anatomy and ultrastructure of palisade-like epidermal cells of *M. sylvatica* faucal scales.** (**A**) Fragment of a longitudinal section of faucal scale with palisade-like epidermal cells and parenchyma. (**B**,**C**) Fragments of palisade-like epidermal cells of faucal scales with visible thin cell wall, numerous mitochondria, plastids, small vacuoles with an osmophilic substance (pink stars), and profiles of smooth endoplasmic reticulum. (**D**) Palisade-like epidermal cell and parenchymal cells. (**E**) Mitochondria with well-developed cristae, secretory vesicles (violet arrow), and dark osmophilic secretion (pink stars) in the cell sap of a palisade-like epidermal cell. (**F**) Fragment of a protoplast of a palisade-like epidermal cell with visible profiles of smooth endoplasmic reticulum, numerous ribosomes, and aggregations of secretory vesicles forming multivesicular bodies; cw—cell wall, mb—multivesicular body, pe—palisade-like epidermal cell, ser—smooth endoplasmic reticulum, m—mitochondrion, n—nucleus, p—plastid, pa—parenchyma, r—ribosome, v—vacuole. Scale bars = 20 µm (**A**), 1 µm (**B**–**D**), 0.5 µm (**E**,**F**).

During anthesis, the palisade-like epidermal cells were characterised by dark-stained protoplasts (Figure 4B–E and Figure 6A). Numerous organelles were observed in the cytoplasm, indicating the high metabolic activity of these cells (Figure 6B–D). Their cell walls were usually thin, except for the outer periclinal wall, which was slightly thicker (Figure 6A). The cell structure revealed small vacuoles (Figure 6B,C), large nuclei (Figure 6D), and numerous organelles, predominantly mitochondria (Figure 6B–E), with well-developed cristae inside. There were also deposits of an osmophilic substance in the vacuoles (Figure 6B,C,E). Plastids observed in these cells were irregularly shaped, had a dark stroma (Figure 6B,C), and contained plastoglobules of various sizes (Figure 6C). Few dictyosomes were observed in the cytoplasm, along with smooth endoplasmic reticulum profiles, numerous ribosomes, and secretory vesicles forming multivesicular bodies (Figure 6F).

**Figure 7:**
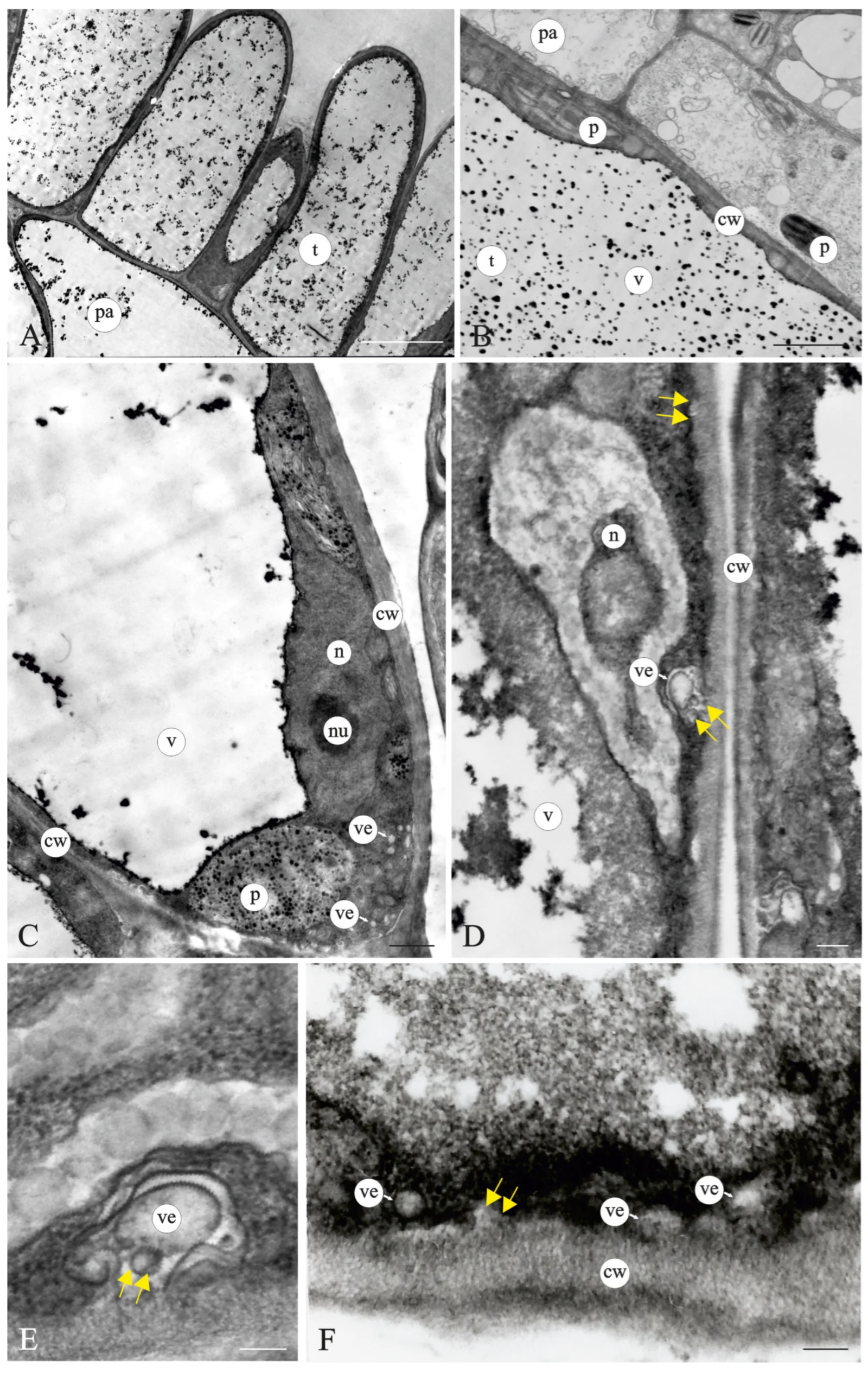
**Ultrastructure of faucal scales trichomes of *M. sylvatica* (TEM).** (**A**) Fragment of a longitudinal section with visible trichomes and parenchymal cells. (**B**,**C**) Parts of trichome cells on the second day of flowering with visible thin cell walls, big vacuoles, nucleus with a nucleolus, plastids, and secretory vesicles. (**D**–**F**) Fragments of trichome cells from flowers on the fourth day of flowering, with visible destruction of protoplasts, a degraded nucleus and plastids, single or clustered secretory vesicles, and secretion (double yellow arrows) in the periplasmic space; cw—cell wall, pe—palisade-like epidermis, n—nucleus, nu—nucleolus, p—plastid, pa—parenchyma, r—ribosome, t—trichomes, v—vacuole, ve—secretory vesicles. Scale bars = 10 µm (**A**,**B**), 2 µm (**C**), 1 µm (**D**), 0.5 µm (**E**,**F**).

**Figure 8:**
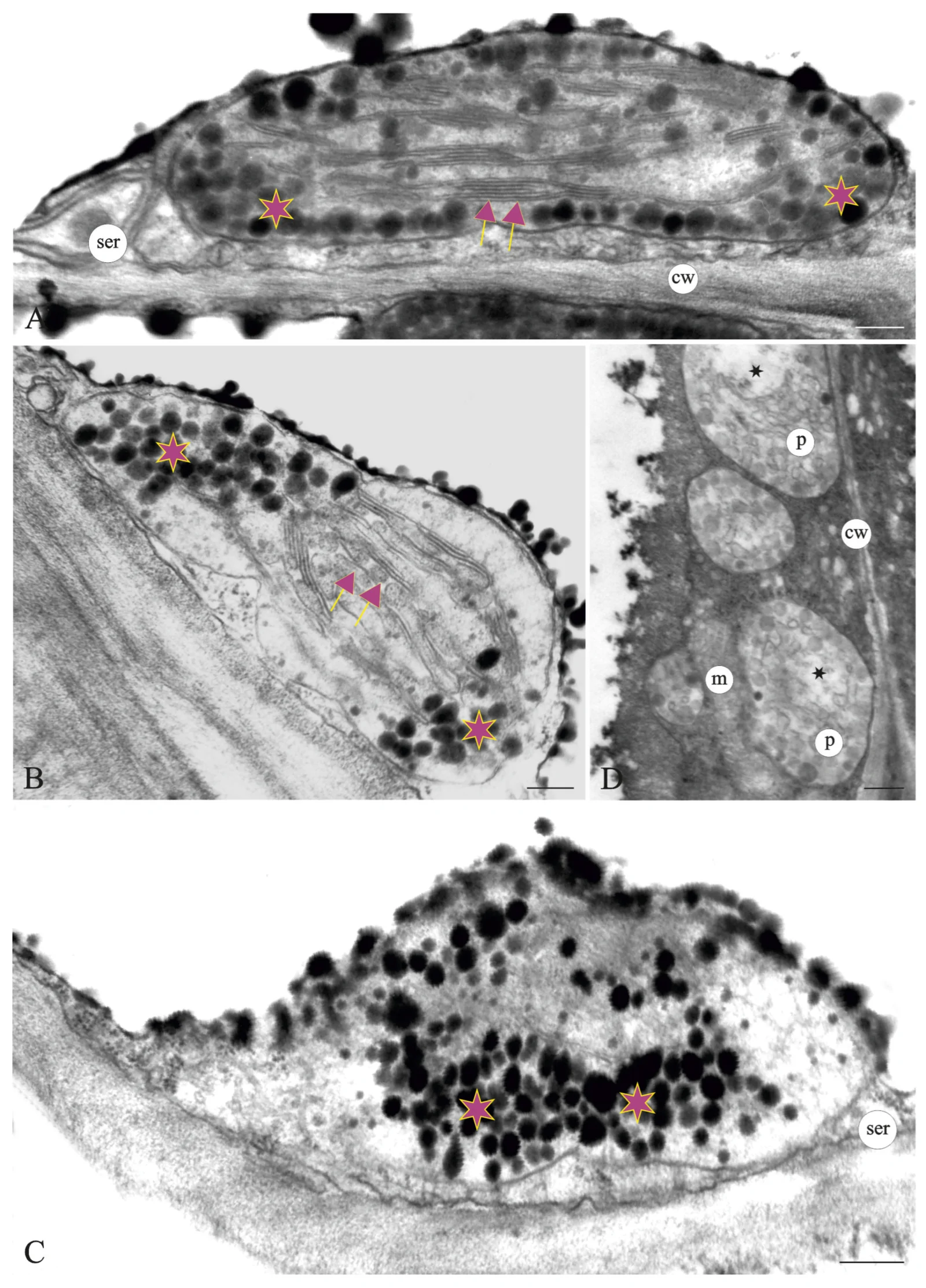
**Parts of trichomes of faucal scales with chromoplasts in *M. sylvatica* on subsequent days of flowering.** (**A**) First day of flowering—protoplast revealing profiles of smooth endoplasmic reticulum, chromoplasts with regular thylakoids (double pink arrows), and numerous plastoglobuli (pink stars). (**B**) Second day of flowering—chromoplast with a partially reduced system of internal membranes and polar-distributed plastoglobules. (**C**) Third day of flowering—chromoplast with visible remnants of membranous structure and numerous plastoglobules. (**D**) Fourth day of flowering—chromoplasts with fragments of membranes from degraded thylakoids and white spaces (black stars) inside the stroma; cw—cell wall, m—mitochondrion, p—plastid, ser—smooth endoplasmic reticulum. Scale bars = 1 µm (**D**), 0.5 µm (**A**–**C**).

A compact arrangement of these unicellular structures was observed in the longitudinal sections of the trichome-covered osmophore zone. Their cell walls were only slightly thicker than those of parenchyma cells (Figure 7A). Plasmodesmata were present in the inner tangential walls adjacent to parenchyma cells. The central part of the trichomes was occupied by a very large vacuole, while the extravacuolar portion of the cytoplasm with organelles was confined to the cell periphery (Figure 7B,C). A distinct nucleolus was clearly visible in the lobed, flattened nucleus (Figure 7C). Chromoplasts contained a system of internal membranes and plastoglobules, with mitochondria (Figure 7B,C) and SER profiles located nearby (Figure 8A,C). In older flowers (fourth day of anthesis, white faucal scales), cellular structures showed signs of degradation. Lighter zones appeared in the nucleus, the tonoplast disintegrated, and flocculent structures were visible in vacuoles (Figure 7D). In turn, individual or clustered secretory vesicles releasing secretion into the periplasmic space were observed in peripheral cytoplasmic regions near the cell walls (Figure 7D–F).

Changes in the plastid structure within trichomes were observed during different flowering stages. On the first day of anthesis, after bud development, the relatively electron-dense stroma revealed regularly arranged internal membranes resembling thylakoids of chloroplasts (Figure 8A). In turn, numerous osmophilic plastoglobules of various electron densities were observed in the peripheral region of chromoplasts and in areas located closer to the central region.

On the second day, the chromoplast stroma became significantly lighter, and the thylakoid system was reduced and restricted to a smaller zone (Figure 8B), with plastoglobules forming clusters concentrated near some polar chromoplasts.

On the third day of anthesis, the thylakoid degradation was even more advanced than on the previous day, with remnants of membrane structures visible near the central region of chromoplasts and numerous plastoglobules surrounding the debris. The polar regions of these plastids were lighter and more electron-lucent (Figure 8C).

In senescent flowers (day 4), the chromoplasts contained fragmented membranes from thylakoid degradation and bright regions of various sizes (Figure 8D).

At all stages of anthesis, electron-dense material resembling the plastoglobuli-forming substance in terms of density was observed in the peripheral region of vacuoles near plastids, which seemed to cross the membranes of the plastids and the tonoplast.

The above observations provide a detailed insight into the anatomical and histochemical characteristics of faucal scales and petal appendages in flowers of two *Myosotis* species, and in the micromorphology of petals. The morphology of different parts of petals was also investigated with particular attention paid to the ultrastructure of faucal scales. Based on the present research findings, it can be concluded that the cells of both yellow faucal scales and white petal appendages exhibit features typical of osmophores.

## 3. Discussion

The present study aimed to characterise the structure of petals and faucal scales in two species, *M. scorpioides* and *M. sylvatica*. Particular attention was paid to the structural organisation of faucal scales, which function as nectar guides. Within these structures, specialised secretory epidermal cells with ultrastructural features typical of secretory cells were identified and interpreted as features consistent with those of osmophores. The findings also indicate that the white ridges present on the petals may serve a similar function.

Noteworthy was also the change in the petal colouration during anthesis, as well as the significance of epidermal texture in pollination ecology. The petal structure, including the faucal scales, was found to be highly similar in both analysed species.

The results of our observations and studies allowed us to distinguish certain features of the analysed species, which are presented in Table 1.

### 3.1. Colour Signals

One of the specific cues typical of the *Myosotis* species is the change in petal colouration during flowering, observed in both the blue lobes and the velvety yellow nectar guides. This phenomenon provides a clear signal to insects, indicating either the presence or absence of nectar in flowers at different stages of anthesis. The appearance of brown, necrotic spots within the nectar guides on the final day of flowering may also be significant for pollinating insects. According to Weiss [45], floral colour changes in *Myosotis* spp. affect specific parts of the flower, namely, the nectar guides, whose colour changes from yellow to white. This author suggested that these colour changes enable insects to distinguish young and rewarding flowers from older and rewardless ones.

The small flowers of *M. scorpioides* and *M. sylvatica* are present in inflorescences, which enhances both the visual display and the scent emission, thereby increasing their attractiveness to pollinators. Previous studies have demonstrated that nectar from flowers of these species is accessible to a broad range of insect groups, including Hymenoptera, Lepidoptera, and Diptera [38]. Observations made by beekeepers indicate that honeybees readily visit *Myosotis* flowers, with an average of four bees recorded per square meter [46].

As reported by Dyer et al. [47] and Lunau et al. [48], bee-pollinated flowers often have blue petals and yellow structures located in the central part of flowers. According to a previous study [38], the petal colour of *M. sylvatica* is perceived as blue not only by humans but also by bees; hence, it may be concluded that *Myosotis* flowers conform to bee colour preferences.

### 3.2. Functions of Papillae on Petals

The adaxial surface of the petals in *M. scorpioides* and *M. sylvatica* is covered with conical papillae. This type of upper epidermal structure is characteristic of petals in approximately 75–80% of plant species [49] and plays a significant role in pollination ecology. Numerous studies have demonstrated the emission and enhanced diffusion of scent molecules to be an important function of papillae [50,51,52]. Flower-secreted odorants most often serve to attract pollinators. They may also protect plants against herbivores and function as a communication route between plants during pest infestations [53,54,55,56].

Conical papillate cells ensure the velvety texture of petals and can enhance their visible pigmentation. This texture can also facilitate pollinator attachment to the petal surface and provide tactile cues that help the pollinator position on the flower [6]. Furthermore, conical epidermal cells contribute to petal reflexing and play a positive role in increasing petal wettability [52,57]. They also focus solar radiation onto absorptive floral pigments, which is essential for floral thermogenesis. The resulting heat may attract pollinators by dispersing volatile compounds [7].

### 3.3. Structure of Putative Osmophores

The histochemical tests (neutral red, Lugol solution, Sudan III, Sudan red B) and the positive sensory test result confirmed that the production and emission of odorant compounds occurs in the yellow faucal scales located in the central part of the flower. Our results largely correspond to literature data on the characteristics of scent-emitting tissues. These include the presence of starch in cells before secretion, the red colouration of vacuoles after neutral red treatment [18,58], and the orange colouration of the protoplast after treatment with Sudan III and Sudan red B [19].

The putative osmophores found in the flowers of the investigated *Myosotis* species were composed of three distinct types of epidermal cells: palisade-like epidermal cells with slightly convex outer walls, papillae, and unicellular trichomes. The palisade-like cells forming osmophores on petals have also been reported in *Anacardium humile* (Anacardiaceae) [27]. In turn, unicellular trichomes have been detected on osmophores in certain Orchidaceae genera, e.g., *Echinosepala* [24] and *Ophrys* [30]. In many plant species, the surface of osmophores located on petals or sepals is formed by papillae, as in some Orchidaceae [22,30], Malpighiaceae [29], and most Bignoniaceae [31], or by papillae and trichomes, as in other Orchidaceae [59] and in *Citrus* [60].

Our observations also reveal the presence of a single vascular bundle with a well-developed xylem, comprising four rows of helical vessels, within each of the five parts of the nectar guides in *Myosotis* flowers. Vogel [18] concluded that abundant vascularisation provides osmophores with sufficient water and sugars for the production and emission of volatile compounds. The direct vascularisation of scent glands in Malpighiaceae was also observed by Posssobom and Machado [29].

### 3.4. Ultrastructure of Osmophore Cells

Similar to the epidermal cells of *Myosotis* faucal scales analysed in the present study, numerous mitochondria and plastids have also been reported in osmophores of other plant species [19,22,25,29,30,61]. A characteristic feature of mitochondria observed in the present study was the presence of well-developed cristae. A similar structure of mitochondria, typical of secretory cells, has been described in osmophores of other species [21,58,62]. The putative osmophore cells of *Myosotis* often showed closely located plastids, mitochondria, and ER profiles, which may indicate the cooperation and intense secretory activity of these structures. A similar organelle configuration has been reported by other authors [19,63,64,65]. Plastids and ER are involved in the synthesis of lipids, being one of the major components of the floral scent [19,66,67,68]. Some other authors have reported that plastoglobules are associated with terpene biosynthesis [62,65]. Also, Gonçalves-Souza et al. [58] have found that the synthesis of volatile compounds in osmophores of *Philodendron adamantinum* occurs within plastids.

Furthermore, multivesicular bodies were observed in the cytoplasm of osmophore cells of *M. sylvatica*, which also indicates their secretory properties. Similar clustered secretory vesicles have been reported in osmophore cells of *Stapelia hirsuta*. These vesicles are responsible for transporting secretions between cells and toward the outer cell walls [19].

Near the cell walls of the putative *Myosotis* osmophores, there were also small, individual secretory vesicles, whose contents were released into the periplasmic space as a result of the fusion with the plasma membrane. The presence of these small secretory vesicles near the plasma membrane has been documented in osmophores of numerous plant species [58,61,69]. Also, pronounced invaginations of the plasmalemma were visible in some regions of the cells of putative *Myosotis* osmophores, which included secretion droplets of various sizes and of different osmophilicity. The released secretion may further accumulate in periplasmic and intercellular spaces [21]. The secretion droplets observed in epidermal osmophore cells subsequently cross the cell wall into the cuticle, where volatilisation of fragrance compounds occurs [64,70,71,72,73]. In the case of scent glands in *Myosotis*, the thin cuticle layer is likely to facilitate this process.

The chromoplasts observed in the secretory trichomes of putative *Myosotis* osmophores were elliptic at the beginning of anthesis and then pleomorphic over time. Similar transformations regarding the shape of plastids in osmophore cells of *Gymnadenia* orchids have been reported by Stpiczyńska [73]. During flowering, a significant reduction in thylakoid membranes occurred in the chromoplasts of *Myosotis* putative osmophores. On the first day of anthesis, a regular thylakoid arrangement with distinct grana was observed, followed by a progressive reduction in the internal membrane system, and only a few membranes remaining on the final day. These changes may be indicative of the rapid degeneration of these organelles during the relatively short lifespan of *Myosotis* flowers (3–4 days).

The chromoplasts found in the *Myosotis* trichome cells contained numerous spherical plastoglobules of various sizes and of different osmophilicity. Similar observations have been reported in Orchidaceae, possessing osmophilic plastoglobules of various electron densities [24]. These authors also reported that plastoglobules were located close to the smooth ER, unlike in the *Myosotis* flowers analysed in the present study.

The high number of plastoglobules, which are associated with terpenoid biosynthesis, present in the chromoplasts during the first days of flowering may indicate the intensive production of volatile fragrance compounds by *Myosotis* flowers. In turn, the progressive degradation of the chromoplast structure occurring on subsequent flowering days, including a reduction in the osmophilicity of plastoglobules, may suggest a gradual decrease in the synthesis of these compounds and termination of their production on the fourth day of flowering, accompanied by the very low electron-density of these structures.

In turn, on all days of anthesis, the osmophore cells of *Myosotis* revealed osmophilic structures resembling plastoglobules located in close proximity to plastids. Similar structures were also found in vacuoles located near the tonoplast. Likewise, in the present study, electron-dense material has been reported in plastids, mitochondria, and outside these structures in the osmophore cells of *Caladium bicolor* [25]. The results reported by these authors suggest that this electron-dense material crossed the membranes of these organelles, which might have also occurred in the *Myosotis* species analysed in the present study, particularly in respect of plastids.

### 3.5. Biochemical Properties and Applications of Myosotis Flowers

Studies of extracts obtained from fresh *M. sylvatica* flowers conducted by Aytar et al. [74] have revealed a high content of total phenolic compounds, including a high proportion of flavonoids (71.8 mg QE/g) and protoanthocyanidins (84 mg CAE/g), indicating potential pharmacological applications of these organs as a natural source of antioxidants. The results of the present histochemical studies (TBO) confirm the high content of phenolic compounds in the vacuoles of the epidermal cells of *M. sylvatica* faucal scales, including trichomes and papillae. The abundance of vacuolar phenolics demonstrated in the *Myosotis* faucal scale epidermal cells analysed in the present study may confirm the inflammatory, antioxidant, and antibacterial properties of *Myosotis* flowers reported by Aytar et al. [74] and valued in ethnopharmacology.

In the flower essential oil of the same species, Aytar et al. [74] detected the presence of 13 volatile compounds. Thymol (37.14%) and carvacrol (10.75%), exhibiting strong antibacterial and antifungal properties, were the dominant constituents. Among the essential oil components, aromadendrene (9.76%), loliolide (5.56%), cymene (5.39%), and chamigrene (4.66%) were also identified. The last three compounds have floral and citrus scents, and their presence is likely to be responsible for the citrus–floral aroma characteristic of *M. sylvatica* flowers, as determined in the sensory test.

Various *Myosotis* species have been used in European herbal medicine since their first use in ancient Greece [75,76,77,78,79,80]. Whole plants (leaves, flowers, and roots) have been used for medicinal purposes. The leaves are applied to heal minor wounds and skin abrasions. The leaf juice is used as a remedy for nosebleeds. The flowers are used to prepare herbal teas or infusions, and are recommended for soothing respiratory conditions, such as coughs and bronchial irritation [81]. Abdu et al. [82] reported anti-inflammatory and antimalarial properties of *M. scorpioides*.

*Myosotis scorpioides* flowers, similar to some other plants, are classified as edible flowers and can be added to dishes to enhance their visual appeal. Since they exhibit biological activities, they promote good health. Their addition to food and supplements is recommended [83].

In summary, based on the results of the anatomical, histochemical, and ultrastructural analyses, as well as the presented photographs, it can be concluded that the yellow faucal scales serving simultaneously as visual nectar guides and the white appendages found on the petals of *M. scorpioides* and *M. sylvatica* flowers function as floral glands producing volatile compounds, i.e., they exhibit features consistent with osmophores. The results of our study have shown for the first time the structure of nectar guides in a representative of the family Boraginaceae. The histochemical tests and cell ultrastructure clearly indicate their secretory properties related to the production and release of essential oil. The present results contribute to expanding the knowledge of the structure and morphological diversity of *Myosotis* osmophores. It can be expected that these results will initiate comparative studies of flowers from other species of the family Boraginaceae, which are characterised by high morphological diversity. The present study, which includes pioneering investigations of Boraginaceae flowers, can provide a basis for further research projects not only encompassing analyses of the floral structures of other species in this family, but also providing insight into their phytochemical properties. Such research may be contribute to the identification of new species with pharmacological applications.

## 4. Material and Methods

### 4.1. Plant Material

Two native species of Polish flora common in natural habitats, *M. scorpioides* and *M. sylvatica*, were selected for this study. *Myosotis scorpioides* most often grows in wet meadows [84], while *M. sylvatica* is found mainly in forests, where it inhabits higher and drier locations; it is also often cultivated for the ornamental value of its flowers [44]. Both taxa exhibit pharmacological traits.

The plant material was obtained in the 2024 growing season from the collection of the Botanical Garden of Maria Curie-Skłodowska University in Lublin, Poland (51°26′20.7″ N, 22°51′37.8″ E). Voucher specimens no. 268 (*M. scorpioides*) and 269 (*M. sylvatica*) were deposited in the Herbarium of the Department of Botany and Plant Physiology, University of Life Sciences in Lublin, Poland.

### 4.2. Sensory Test of Flowers

Freshly opened *M. sylvatica* flowers collected from 20 plants were subjected to sensory assessment, which involved the excision of faucal scales (10 sealed plastic vials) and the remaining petal parts (10 sealed plastic vials), which were subsequently placed in different plastic containers. Each plastic vial containing elements from 30 flowers was sealed tightly and kept at room temperature (23 °C). Five plastic vials from each group were opened after 30 min, whereas the remaining five were checked after 12 h and then after 24 h. The three-fold attempt to open the plastic vials was prompted by the absence of a detectable odour after 30 min and 12 h, as reported by the testers. The scent became apparent only after the flowers were kept in the vials for a longer period (after 24 h). The assessment took place in an odour-free room with good ventilation and no competing food or cleaning smells. Six different people (three students of the University of Life Sciences in Lublin at the age of 20 years (a woman), 21 years (a woman), and 22 years (a man), and three researchers from the Department of Botany and Plant Physiology at the age of 48 years (a man), 52 years (a woman), and 55 years (a woman); all non-smokers and not experiencing respiratory difficulties) were instructed on the principles of the analysis before a blind evaluation of each container, determining the intensity and type of the scent. The participants of the sensory test were instructed not to use perfumes and body or food deodorizers. Two testers sniffed one plastic vial containing faucal scales and one vial containing the remaining parts of the *Myosotis* flower corolla. In turn, the other four participants (who declared to be sensitive to odours) tested two samples containing faucal scales and two samples containing the remaining parts of the corolla. Each tester sniffed each sample three times at five-minute intervals. Odour strength was determined using a five-point scale, as follows: 0—no scent, 1—barely perceptible scent, 2—light scent, 3—moderately intense scent, 4—relatively strong scent, 5—very intense scent. The type of scent was determined by assigning it into one of the following seven categories: (a) fruity, (b) citrusy-floral, (c) herbal, (d) woody, (e) sweet, (f) decayed/fermented (g) grassy, (h) other. The results were expressed as arithmetic means.

### 4.3. Identification of Osmophores

Flowers were collected from 20 randomly selected plants (on average one flower was collected from each plant) on the successive days of anthesis (days 1–4; 20 flowers from each day of life). From the collected material, 5 flowers from successive days of life (total *n* = 20) were selected for SEM observations, 5 flowers were intended for TEM examination (total *n* = 20), and 10 flowers were used for light microscopy examination (in total *n* = 40). Additionally, preliminary observations of floral morphology (*n* = 10 from successive days of anthesis) were performed using a stereoscopic microscope type ZX16 coupled with a Nikon Coolpix 4500 digital camera (Nikon, Tokyo, Japan) and relevant photographs were taken. For locating fragrance-producing tissues, the flowers were embedded in an aqueous solution of 0.1% neutral red for 24 h [18] and then removed and observed.

#### 4.3.1. Scanning Electron Microscopy (SEM)

For surface examination, fresh flowers (*n* = 5) and their petals (*n* = 10) and faucal scales (*n* = 10) were fixed in a 2.5% glutaraldehyde solution in 0.1 M sodium phosphate buffer (pH 7.0) for 2 h at room temperature. Afterwards, the samples were washed in phosphate buffer (4× at 20-min intervals) and dehydrated in increasing concentrations of ethanol (POCH, Gliwice, Poland)—30%, 50%, 70%, 90%, 96%, and 100% (3× for 30 min). In the next step, the plant material was critical-point-dried in liquid CO_2_ using a K 850 critical point dryer (Emitech, Laughton, East Sussex, UK) and sputter-coated with gold (20 nm thickness) [85] using a K 550X device (Emitech, Ashford, UK). The observations were made with a Tescan Vega II LMU scanning electron microscope (Tescan, Brno, Czech Republic) at an accelerating voltage of 30 KeV. Images were taken using a SE detector and saved using Vega software ver. 3.5.19.

#### 4.3.2. Light Microscopy (LM)

Fresh flowers were used for light microscopy, from which hand-made transverse and longitudinal sections were prepared with a razor blade in the region of the faucal scales. Some unstained sections were used to prepare semi-permanent slides in a glycerol–water solution (1:1, *v*/*v*) and used as a control. A portion of the material was used for the detection of starch grains using Lugol solution [86], total lipids using Sudan III [87] and Sudan Red B [88], phenolic compounds using Toluidine blue O (pH 4.5) [89,90], and scent-secreting tissue using neutral red [18].

Semi-thin sections of flowers and fragments of petals with faucal scales were prepared from the flowers at four stages of development (1-, 2-, 3-, and 4-day flowers, with 10 flowers in each stage). The plant material was fixed in 2.5% glutaraldehyde in phosphate buffer (pH 7.2; 0.1 M) at 4 °C for 24 h. Half of the material from each sample was used for TEM, and the remaining samples were washed three times in phosphate buffer, dehydrated in an ethanol series (15, 30, 50, 70, 90, 96, and 99.8%) and embedded in LR white resin (LR white acrylic resin, medium grade, Sigma-Aldrich, Saint-Louis, MO, USA). Tissue blocks were cut transversely and lengthwise (0.7–0.9 µm thick) using a Reichert Ultracut S ultramicrotome (C. Reichert Optische Werke AG, Vienne, Austria) and a glass knife, and stained in 1% methylene blue with 1% azure B in a 1% aqueous sodium tetraborate solution [91].

#### 4.3.3. Transmission Electron Microscopy (TEM)

Plant samples, processed in accordance with the procedure described above, were fixed in glutaraldehyde and post-fixed with a 1% osmium tetroxide solution (0 °C for 1.5 h) and washed three times in distilled water. Subsequently, they were dehydrated in a graded ethanol series and embedded in LR white resin (LR white acrylic resin, medium grade, Sigma-Aldrich). Following polymerisation at 60 °C, ultra-thin sections (60–70 nm thickness) were cut using a Reichert Ultracut S ultramicrotome and a glass knife. In the last step, the plant material was stained with 0.5% uranyl acetate and post-stained with 0.5% lead citrate [92]. Observations were made with a JEM 1400 (JEOL Cp., Tokyo, Japan) transmission electron microscope at an accelerating voltage of 120 kV. Photographic documentation was performed using an 11 Magapixel TEM Camera MORADA G2 (EMSIS GmbH, Münster, Germany).

Hand-made and semi-thin slides were examined and photographed under an Olympus CX23 light microscope (Olympus, Tokyo, Japan) equipped with an Olympus EP50 digital camera (Olympus, Tokyo, Japan) and EPview software (ver. 1.2.4.2.2).

## 5. Conclusions

- Faucal scales and petal appendages located in the central part of the flower may function as nectar guides due to their contrasting yellow colour and surface texture, and as putative osmophores due to scent emission.
- Both the upper petals and the surfaces of faucal scales and petal appendages exhibit features typical of secretory cells involved in the production and emission of volatile compounds.
- The study showed morphological differences in faucal scale rings between the studied *Myosotis* species. These segments were bilobate in *M. sylvatica* and undivided in *M. scorpioides*, a feature that may have taxonomic significance.
- This study presents for the first time the structures of putative osmophores in a representative of the family Boraginaceae, which may initiate further studies of scent glands in flowers of other members of this family.
- The results of the present histochemical studies, indicating high levels of phenolic compounds in the epidermal cells of faucal scales, may encourage further ethnopharmacological research.

## Figures and Tables

**Table 1:** Comparison of features of the analysed *Myosotis* species.

Feature	*M. scorpioides*	*M. sylvatica*
**Period of flowering**	V–IX	IV–VI
**Calyx indumentum**	Adherent, upwardly curved trichomes	Protruding, hooked trichomes
**Corolla**	Faucal scales undivided	Faucal scales bilobate
**Petal anatomy**	No differences between the species	

## Data Availability

The datasets used and/or analyzed during the current study are available from the corresponding author on reasonable request.
